# Study of the Interfacial Bond Behavior between CFRP Grid–PCM Reinforcing Layer and Concrete via a Simplified Mechanical Model

**DOI:** 10.3390/ma14227053

**Published:** 2021-11-20

**Authors:** Huijuan Dai, Bo Wang, Jiawei Zhang, Junlei Zhang, Kimitaka Uji

**Affiliations:** 1 School of Civil Engineering, Xi’an University of Science and Technology, Xi’an 710054, China; daihuijuan1985@163.com; 2 School of Civil Engineering, Chang’an University, Xi’an 710061, China; zaq197125zaq@163.com; 3 Department of Civil and Environmental Engineering, Tokyo Metropolitan University, Tokyo 192-0397, Japan; zhangjunlei2009@yahoo.co.jp (J.Z.); k.uji@tmu.ac.jp (K.U.)

**Keywords:** CFRP grid, PCM, interface, mechanical model, pull-out test, finite element analysis

## Abstract

This paper presents the results of pull-out tests conducted to investigate the interfacial bond behavior between a carbon-fiber-reinforced polymer (CFRP) grid–polymer cement mortar (PCM) reinforcing layer and existing concrete, and proposes a simplified mechanical model to further study the interface bond mechanism. Four specimens composed of a CFRP grid, PCM, and concrete were tested. The influence of the type of CFRP grid and the grid interval on the interface bond behavior was discussed. The failure patterns, maximum tensile loads, and CFRP grid strains were obtained. The change process of interface bond stress was investigated based on the grid strain analysis. In addition, the simplified mechanical model and finite element model (FEM) were emphatically established, and the adaptability of the simplified mechanical model was validated through the comparative analysis between the FEM results and the test results. The research results indicate that a CFRP grid with a larger cross-sectional area and smaller grid interval could effectively improve the interface bond behavior. The tensile stress was gradually transferred from the loaded edge to the free edge in the CFRP grid. The interface bond behavior was mainly dependent on the anchorage action of the CFRP grid in the PCM, and the bond action between the PCM and the concrete. The FEM results were consistent with the test results, and the simplified mechanical model with nonlinear springs could well describe the interface bond mechanism between the CFRP grid–PCM reinforcing layer and concrete.

## 1. Introduction

**Figure 1:**
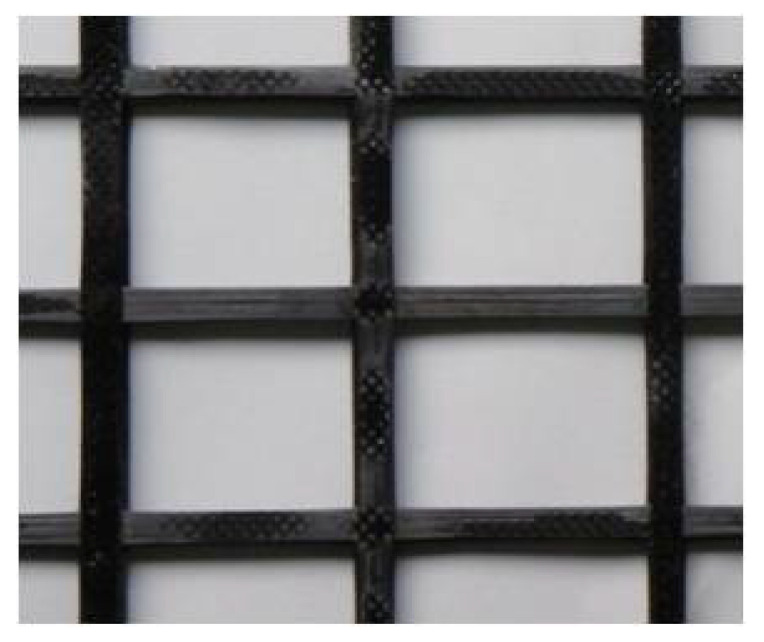
CFRP grid.

**Figure 2:**
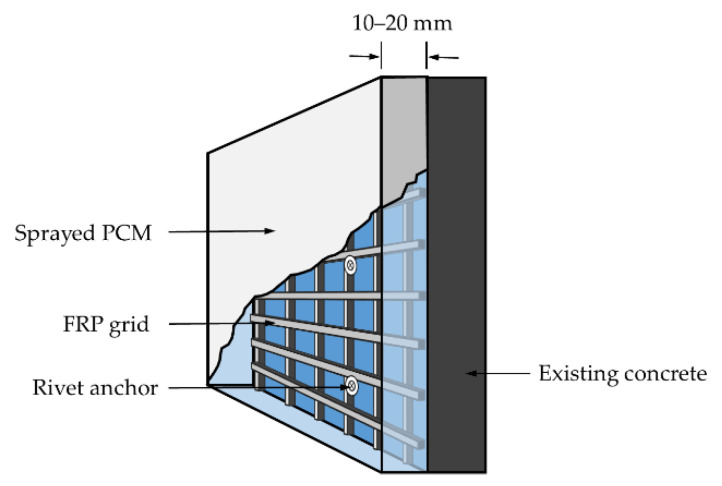
Schematic diagram of strengthened concrete structures using CFRP grid and PCM.

Carbon-fiber-reinforced polymer (CFRP) has been widely used in the repair and strengthening of concrete structures, because of its favorable and prominent properties (e.g., high strength-to-weight ratio, excellent corrosion resistance, and durability) [1,2,3]. Recently, a new strengthening technique for concrete structures using CFRP grids and sprayed PCM has caught the attention and interest of the civil engineering realm [4,5,6,7]. CFRP grid is made from continuous impregnated high-strength carbon fiber roving alternating in both longitudinal and transverse directions to form a cross-laminated grid structure with a certain rigidity, as shown in Figure 1; it has unique mechanical characteristics compared to other CFRP composites (e.g., CFRP sheets, CFRP plates and strips); that is, the horizontal grids and vertical grids jointly resist the external loads applied on the CFRP grid. Some investigations have found that CFRP grid has a satisfactory strengthening effect in improving the mechanical performance of existing concrete structures, including beams, slabs, and columns in concrete member, as well as large-span structures such as bridges and tunnels [8,9,10,11]. In addition, CFRP grid can be applied to strengthen underwater structures, because of its good bond performance with existing concrete in low-temperature and moist environments [8,12]. Currently, FRP sheets and plates are mainly used as the strengthening materials in the FRP-strengthening technique. However, there are a few drawbacks for the FRP sheets/plates jacketing method, such as the insufficient bond strength between FRP sheets/plates and existing concrete in wet environments. For this new strengthening method, the drawbacks can be effectively avoided by riveting the CFRP grid to the concrete surface. Moreover, as a protective (thermal insulation) system for CFRP grid, PCM significantly improves the peeling resistance, fire resistance, and durability [5,6,13]. PCM, a new type of modified mortar, is formed by adding a certain content of organic polymer to the conventional cement mortar; it has some advantages over the conventional cement mortar, such as high tensile strength, low elastic modulus, favorable impermeability, and frost resistance, as well as excellent bond behavior with the existing concrete [5,6,14,15]. In this new strengthening method with CFRP grid and sprayed PCM, the CFRP grid is installed on the surface of existing concrete via a rivet anchor, and PCM is sprayed on the outside surface of the CFRP grid and concrete. By means of this construction technique, the CFRP grid–PCM reinforcing layer and the existing concrete structure would be given the integrity to resist external force [16,17,18,19,20,21], which could improve the bearing capacity, stiffness, and durability of existing concrete structures, as shown in Figure 2. Compared with the conventional strengthening method using steel or FRP plates for jacketing, this new strengthening method with CFRP grid and a PCM reinforcing layer has some technical advantages, including lesser thickness of the reinforcing layer (10–20 mm), more convenient construction, lower costs and environmental dependence, etc. [5,6].

In the past few years, some investigators have studied the effectiveness of CFRP grid in strengthening concrete structures, indicating that this strengthening method could significantly improve the mechanical behavior of concrete structures. Nevertheless, the tensile strength of CFRP grid has not been fully utilized, due to premature interface debonding of the reinforcing layer [5,6,13,22,23]. Therefore, it is clear that the reliable bond behavior between the CFRP grid, PCM, and concrete is the key to giving full play to the high tensile strength of CFRP grid, and improving the strengthening efficiency. Guo et al. [5,6] investigated the influence of parameters such as the number of grid points, grid interval, and type of PCM on the interface bond behavior, indicating that the interface peeling failure occurs when the tensile stress exceeds the ultimate bond strength between PCM and concrete. Ding et al. [17] conducted a pull-out experiment focusing on the effects of the embedded length of CFRP grid and horizontal grid on the bond behavior of the CFRP grid–concrete interface, and found the critical embedded length required to make full use of the tensile strength of the CFRP grid. Sugiyama et al. [24] tested the interfacial bond strength between the CFRP grid–PCM reinforcing layer and concrete, showing that the type of PCM has no effect on the bond strength. However, considering that the interface between the CFRP grid–PCM reinforcing layer and concrete consists of three materials, and the PCM–concrete interface is divided into a checkerboard pattern by CFRP grid, resulting in a complicated stress state of the interface, experiments alone are not enough to reveal the bond mechanisms between CFRP grid, PCM, and concrete in detail, and further investigations based on mechanical analysis and numerical simulation are needed—similar to the research of N.K. Banjara et al. [25]. Dung [26] studied the shear bond behavior of the interface between concrete substrate and repaired mortar by combining test and finite element analysis; however, the analysis did not consider the role of grid points, and the analysis content was not comprehensive enough.

In this study, pull-out tests of four plate-type specimens with CFRP grid–PCM reinforcing layer and concrete were conducted to ascertain the maximum tensile loads and failure modes corresponding to different CFRP grid types and grid intervals. Based on the experimental results, the preliminary investigation of the bond mechanism between concrete and the CFRP grid–PCM reinforcing layer was carried out via strain analysis of the CFRP grid. Compared with the existing research, interfacial bond strength between the CFRP grid–PCM reinforcing layer and concrete was quantified via strain analysis. In addition, a simplified analysis model was proposed based on mechanical analysis. Compared with the existing models, the proposed model is more convenient to apply. Finally, in order to verify the accuracy of this proposed model, a two-dimensional finite element (FEM) analysis was conducted, putting forward a new method for the follow-up study of concrete strengthened by CFRP grid–PCM.

## 2. Experimental Program

### 2.1. Details of Specimens

**Figure 3:**
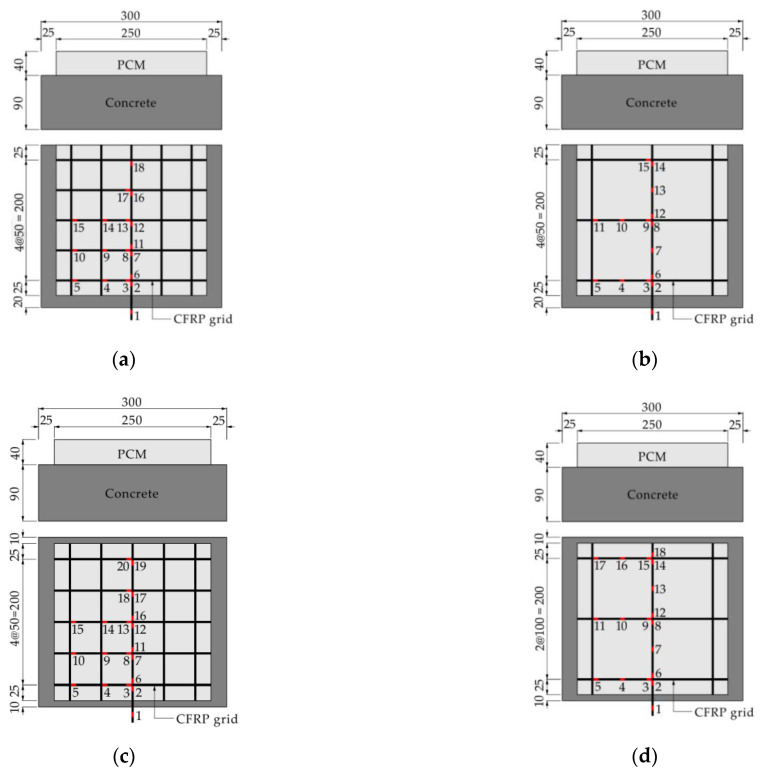
Dimensions of the specimens and arrangement of strain gauges on the CFRP grid. (**a**) C6D50; (**b**) C6D100; (**c**) C8D50; (**d**) C8D100 (units: mm).

Four plate-type specimens composed of concrete, CFRP grid, and PCM were fabricated. The investigation variables included the type of CFRP grid (CR6 and CR8) and the grid interval (50 mm and 100 mm). Table 1 summarizes the details of all specimens. Figure 3 shows the specimens’ geometry and the arrangement of the strain gauges. The production process of the specimens can be summarized as three stages: When the curing time of concrete in a moist environment reached 21 days, the concrete surface was treated via vacuum blast. After 28 days, the CFRP grid was installed on the concrete surface by coating with epoxy primer and spraying PCM with a thickness of 40 mm. Finally, pull-out tests were carried out after the age of concrete reached 46 days. Table 2 and Table 3 show the mix proportions of the concrete and PCM, respectively. The material properties of the concrete and PCM are summarized in Table 4. The material properties of the CFRP grid are shown in Table 5.

### 2.2. Load Program

**Figure 4:**
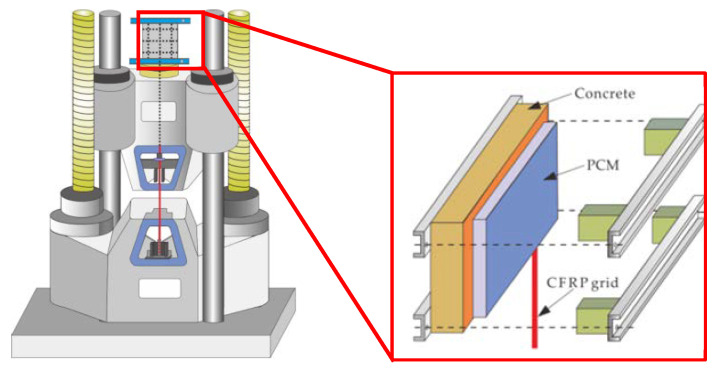
Diagram of specimen loading.

Pull-out tests were conducted based on JSCE-E 539-2007 [27] and ACI 440.1R-06 [28]. The monotonic tensile load with a loading rate of 200 N/mm^2^/s was subjected to a vertical CFRP grid extended from the bond interface. To prevent eccentricity loads during the loading process, four iron blocks and steel plates were used to fix the position of the specimens. The strain gauges were installed on the surface of the CFRP grid to measure the strain values of the CFRP grid. The test schematic is shown in Figure 4.

## 3. Experimental Results and Discussion

### 3.1. Failure Patterns and Maximum Loads

**Figure 5:**
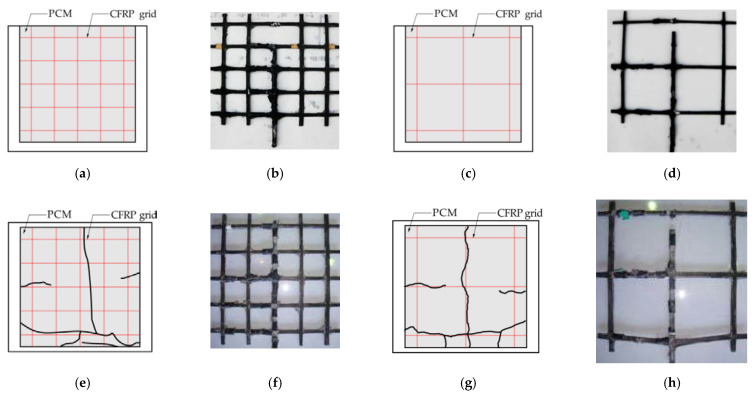
Failure patterns of the specimens: (**a**) failure pattern of PCM for specimen C6D50; (**b**) failure pattern of CFRP grid for specimen C6D50; (**c**) failure pattern of PCM for specimen C6D100; (**d**) failure pattern of CFRP grid for specimen C6D100; (**e**) failure pattern of PCM for specimen C8D50; (**f**) failure pattern of CFRP grid for specimen C8D50; (**g**) failure pattern of PCM for specimen C8D100; (**h**) failure pattern of CFRP grid for specimen C8D100.

Figure 5 shows the failure patterns of all specimens. The loading position is at the bottom of the sketch. It can be observed that the vertical tensile grids in all specimens were pulled out, and PCM developed surface cracks to varying degrees, while the interface between the PCM and the concrete remained intact throughout the whole loading process. This could attributable to the fact that epoxy primer was used on the surface between the CFRP grid–PCM reinforcing layer and the concrete, resulting in the improvement of interface bond action. For the specimens C6D50 and C6D100, no cracks appeared on the surface of the PCM, and the specimens failed due to the vertical tensile grids being pulled out. For the specimens C8D50 and C8D100, the vertical tensile grids were pulled out, and cracks appeared on the PCM. It can be seen that the degree of damage to the CFRP grids for specimens C6D50 and C6D100 was more severe than that for specimens C8D50 and C8D100. This might be attributable to the fact that the ultimate tensile action (24.5 kN) of the CR6-type CFRP grid used in specimens C6D50 and C6D100 was lower than that (36.96 kN) of the CR8-type CFRP grid used in specimens C8D50 and C8D100. Table 6 shows the maximum loads and failure patterns of all specimens. The tensile strength utilization ratio of CFRP was defined as the ratio of the maximum tensile load of the specimens to the test ultimate tensile action of the CFRP grids [29]. The tested ultimate tensile loads of the CR8 and CR6 CFRP grids were 45.07 kN and 28.6 kN, respectively. It can be seen that the tensile strength utilization ratio of the CFRP grid in specimen C6D50 was 84% higher than that in specimen C6D100, and the tensile strength utilization ratio of the CFRP grid in specimen C8D50 was 23% higher than that in specimen C8D100, indicating that the tensile strength utilization ratios of the CFRP grid with small grid intervals were higher than those with large grid intervals. This might be attributable to the fact that the smaller grid interval has more grid points to resist the tensile load, resulting in the higher tensile strength utilization ratio of the CFRP grid for specimens with smaller grid intervals compared to the specimens with larger grid intervals. In addition, it can be seen that the maximum load of specimen C8D50 with the CR8@50-type CFRP grid was 133% higher than that of specimen C6D50 with the CR6@50-type CFRP grid, and the maximum load of specimen C8D100 with the CR8@100-type CFRP grid was 247% higher than that of specimen C6D100 with the CR6@100-type CFRP grid, indicating that the specimen with the larger cross-sectional area of the CFRP grid could obtain a higher tensile capacity under the precondition of reliable interface bonding. Meanwhile, comparing specimens C6D100 and C8D50 showed that the simultaneous use of a CFRP grid with a larger cross-sectional area and smaller grid interval can significantly improve the interfacial bearing capacity between the CFRP grid–PCM reinforcing layer and the concrete.

### 3.2. Strain Distribution of CFRP Grids

**Figure 6:**
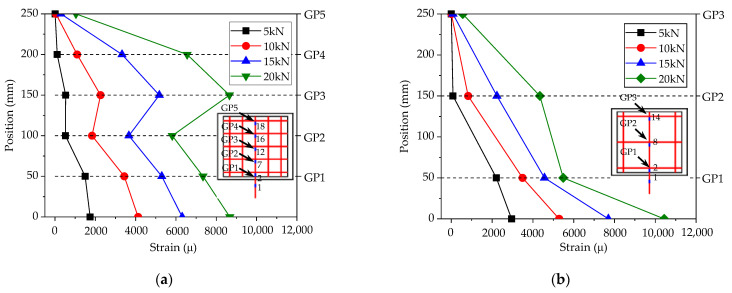
Strain distribution patterns of the vertical grid. (**a**) C8D50; (**b**) C8D100.

According to the analysis above, the stress transfer mechanism between the CFRP grid–PCM reinforcing layer and the concrete is rather complicated. So as to preliminarily investigate the mode of tensile stress transfer between the three materials, the specimens C8D50 and C8D100 were taken as examples to analyze the strain distribution of the vertical tensile CFRP grid, as shown in Figure 6. It can be observed that the strain on the vertical tensile grid gradually decreased from the loaded edge to the free edge at the lower load level. However, the strain distribution patterns changed at the higher load level. For the specimen C8D50, as shown in Figure 6a, the strain of grid point GP3 sharply increased and exceeded the strain of grid point GP2 after the tensile load reached approximately 10 kN. This might be attributable to the fact that the restraining action for grid point GP3 from PCM gradually decreased with the increase in the tensile load due to the fracture of PCM (see Figure 5e), resulting in a change in the tensile stress transfer mode of the CFRP grid. For the specimen C8D100, as shown in Figure 6b, when the tensile load exceeded approximately 15 kN, the strain of grid point GP2 increased sharply, while the strain of grid point GP1 increased slightly, indicating that under the higher load, the PCM near the loaded edge fractured severely, and lost its restraining action on the grid points; after this, the tensile load was transferred to other grid points, where the PCM was relatively intact or only slightly fractured. In addition, comparing the strain distributions of two specimens, it can be found that the strain difference between the vertical grid near the loaded edge and the vertical grid far away from the loaded edge for specimen C8D50 was smaller than that for specimen C8D100, indicating that the strain distribution of the vertical grid in specimen C8D50 was more uniform than that in specimen C8D100. This might be explained by the fact that the grid interval of the CFRP grid in specimen C8D50 was smaller than that in specimen C8D100, resulting in more grid points in specimen C8D50 to share the tensile load compared to specimen C8D100.

## 4. Interface Bond Mechanism

### 4.1. General

**Figure 7:**
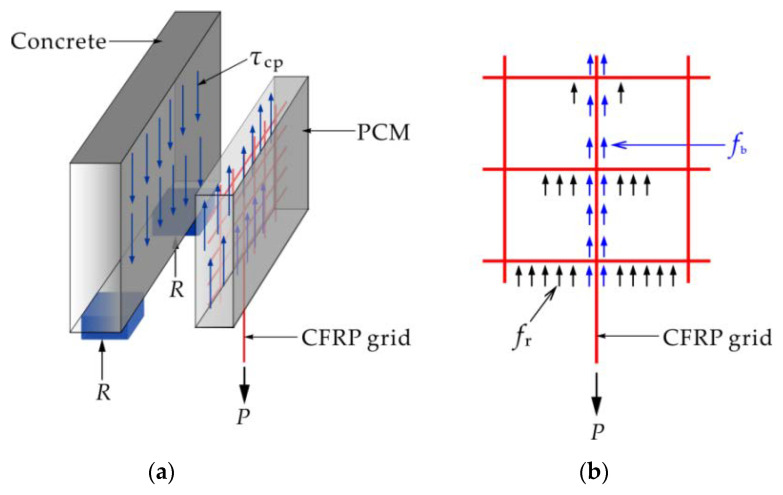
Schematic diagram of the stress transfer process for the specimen: (**a**) interface bond stress between the CFRP grid–PCM reinforcing layer and the concrete; (**b**) anchorage action of the CFRP grid in the PCM. *τ*_cp_: bond stress between the reinforcing layer and the concrete; *f*_b_: bond action between the vertical grid and the PCM; *f*_r_: resistant action of the horizontal grid.

The tensile stress transfer process between the CFRP grid–PCM reinforcing layer and the concrete can be divided into two stages, as shown in Figure 7. In the first stage, the tensile load applied to the vertical CFRP grid was transferred to the PCM via the anchorage action of the CFRP grid in the PCM, as shown in Figure 7b. In the second stage, the tensile stress borne by the PCM was transferred to the concrete via the interface bond between the CFRP grid–PCM reinforcing layer and the concrete, as shown in Figure 7a. Based on the experimental results mentioned above, it can be seen that the epoxy primer as an interfacial binder could effectively improve the bond behavior between the CFRP grid–PCM reinforcing layer and the concrete. Similarly, the failure modes of the specimens mainly depended on the CFRP grid and the PCM. So as to make full use of the high tensile strength of the CFRP grid, a credible anchorage of the CFRP grid in the PCM must be ensured. Previous investigators have noted that the anchorage action of the CFRP grid in PCM is mainly provided by two parts: the resistant action of the horizontal grid, and the bond action of the vertical grid [30]. In addition, it is clear that the tensile stress transfer mode between the CFRP grid and the PCM changes with the generation of cracks on the surface of the PCM. Consequently, the specimen C8D100 was taken as an example to reveal the stress behavior of the CFRP grid in PCM in further detail, based on the strain analysis of the CFRP grid.

### 4.2. Load–Strain Difference Curves

**Figure 8:**
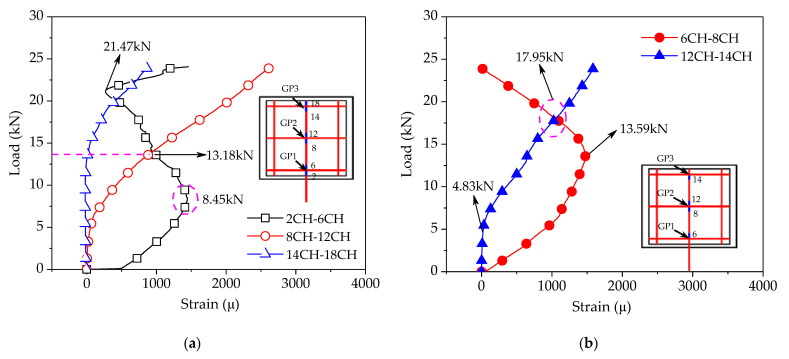
Load–strain difference curves of the grid points: (**a**) strain difference of the grid points; (**b**) strain difference in grid intervals.

As discussed previously, the interface bond between CFRP grid–PCM reinforcing layer and concrete can be significantly improved by using the epoxy primer, and the tensile bearing capacity of the specimen is determined by the anchorage action of the CFRP grid in the PCM. So as to further clarify the anchorage action of the CFRP grid in the PCM, the resistant action of the horizontal grid and bond action of the vertical grid were characterized based on the strain difference on both sides of the grid points for the vertical grid, and the strain difference between the bottom and top ends within the grid interval, as shown in Figure 8. The strain values can be measured by strain gauges arranged on the CFRP grid. It was found that, under the lower load, with the increase in the tensile load, the strain difference of grid points near the loaded edge sharply increased, while the strain difference of the grid points far away from the loaded edge increased slightly. This indicates that the tensile stress was transferred from the loaded edge to the free edge by the resistant action of the horizontal grid at the grid points. However, under the higher load, the tensile stress transfer mode changed. As shown in Figure 8a, the strain difference ε (2CH)–ε (6CH) of grid point GP1 gradually decreased after the tensile load exceeded 8.45 kN, while the strain differences of the remaining grid points continued to increase with the increase in the tensile load. Past a load of 13.18 kN, the strain difference of grid point GP1 was lower than the strain difference ε (8CH)–ε (12CH) of grid point GP2. This phenomenon might be attributable to the fact that the PCM at grid point GP1 fractured, which reduced the resistant action of grid point GP1, resulting in the transfer of tensile stress from grid point GP1 to grid points GP2 and GP3. Accordingly, the resistant action of grid points GP2 and GP3 gradually increased. In addition, it can be found that the strain difference of grid point GP1 began to increase after the tensile load reached 21.47 kN. This might be caused by the pulling out of the vertical grid (2CH) near the loaded edge. As shown in Figure 8b, under the lower load, with the increase in the tensile load, the increase in strain difference within the grid interval near the loaded edge was more significant than that far away from the loaded edge—especially the strain difference ε (12CH)–ε (14CH) far away from the loaded edge, which began to increase until the tensile load reached ~4.83 kN. This indicates that the tensile stress was transferred from the loaded edge to the free edge by the bond action between the vertical grid and the PCM. However, under the higher load (i.e., 13.59 kN), with the increase in the tensile load, the strain difference ε (6CH)–ε (8CH) near the loaded edge gradually decreased, while the strain difference ε (12CH)–ε (14CH) far away from the loaded edge continued to increase. When the tensile load exceeded 17.95 kN, the strain difference ε (12CH)–ε (14CH) was larger than the strain difference ε (6CH)–ε (8CH). This might be attributable to the fact that the fracture of the PCM near the loaded edge reduced the bond action of the vertical grid, resulting in the transfer of tensile stress via the bond action of the vertical grid far away from the loaded edge.

## 5. Simplified Interface Model

### 5.1. Simplified Mechanical Model

**Figure 9:**
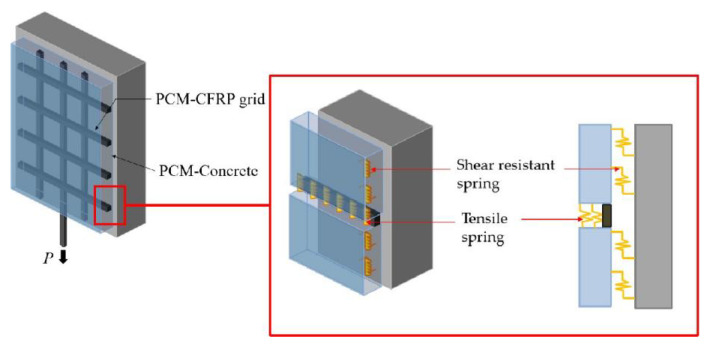
Schematic diagram of the specimen interface.

According to the analysis of the interface bond mechanism between the CFRP grid–PCM reinforcing layer and concrete, the tensile stress is balanced by the anchorage action of the CFRP grid in the PCM and the interface bond action between the CFRP grid–PCM reinforcing layer and the concrete. Therefore, a simplified model based on a nonlinear spring system was proposed from the perspective of mechanical analysis, as shown in Figure 9. It is clear that the resistant action of the horizontal grid plays a dominant role in the anchorage action of the CFRP grid in the PCM, so the tensile springs and shear-resistant springs are used to simulate the resistant action of the CFRP grid in the PCM and the interface bond action between the reinforcing layer and the concrete, respectively. In order to reduce the complexity of the model, the two categories of springs are equivalent to the same category of spring in terms of stiffness.

**Figure 10:**
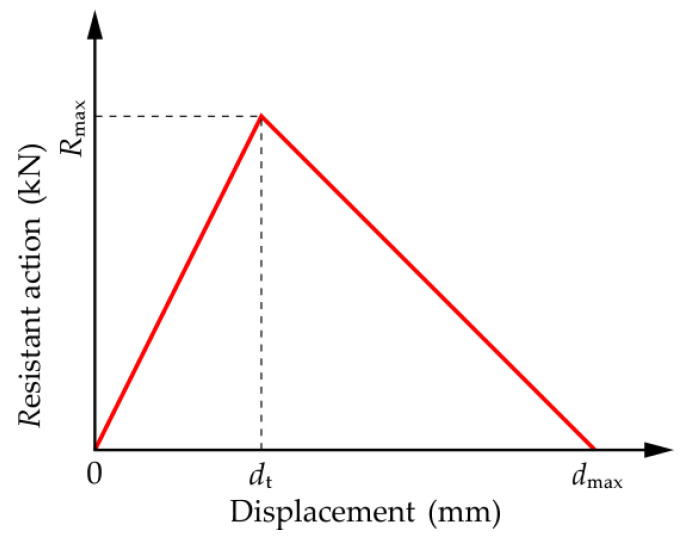
Stiffness curve of the nonlinear spring.

In view of the brittle failure characteristics of specimens, a nonlinear spring was used for the equivalent simulation. Figure 10 shows the load–deflection curve of the nonlinear spring. The load–deflection curve can be divided into two stages: In the first stage, the resistant action of the spring increased linearly with the increase in deflection until the spring deflection reached the critical deflection *d*_t_. When the spring deflection was equal to the critical deflection *d*_t_, the resistant action of the spring reached the maximum value *R*_max_. In the second stage, the spring deflection increased rapidly and resistant action gradually decreased after the spring deflection exceeded the critical deflection *d*_t_. When the spring deflection reached the maximum value *d*_max_, the resistant action of the spring was equal to zero. At this point, the spring failed, and the stress was transferred to the adjacent springs until all springs failed, and the spring system lost its bearing capacity.

The critical deflection *d*_t_ and maximum deflection *d*_max_ of the nonlinear spring were determined by the concrete material test, and the maximum resistant action *R*_max_ was determined by the interface shear strength between the PCM and concrete, along with the tensile strength of the PCM. Each spring represents the local interface shear area and tensile area of the PCM. The bearing capacity of the nonlinear spring can be calculated by Equation (1):

(1) $$R_{\max}=\left(R_{s}+R_{t}\right)\times \beta \;R_{s}=h\times s\times \sigma_{s}\;R_{t}=t\times s\times \sigma_{t}$$

where *β* is the adjustment coefficient; *s* is the spring spacing; *t* is the PCM thickness; *h* is the horizontal grid interval; *σ*_s_ is the interface shear strength between the PCM and the concrete; and *σ*_t_ is the tensile strength of the PCM.

### 5.2. Finite Element Analysis

In order to conduct a further investigation of the interface bond mechanism and tensile stress transfer mode between the CFRP grid–PCM reinforcing layer and the concrete, the FEM analysis was carried out on the basis of pull-out tests. At first, the effectiveness of the simplified model was verified via the numerical simulation method. Then, the stress variation of the CFRP grid was explored based on the results of the FEM analysis.

#### 5.2.1. Establishment of the Model

**Figure 11:**
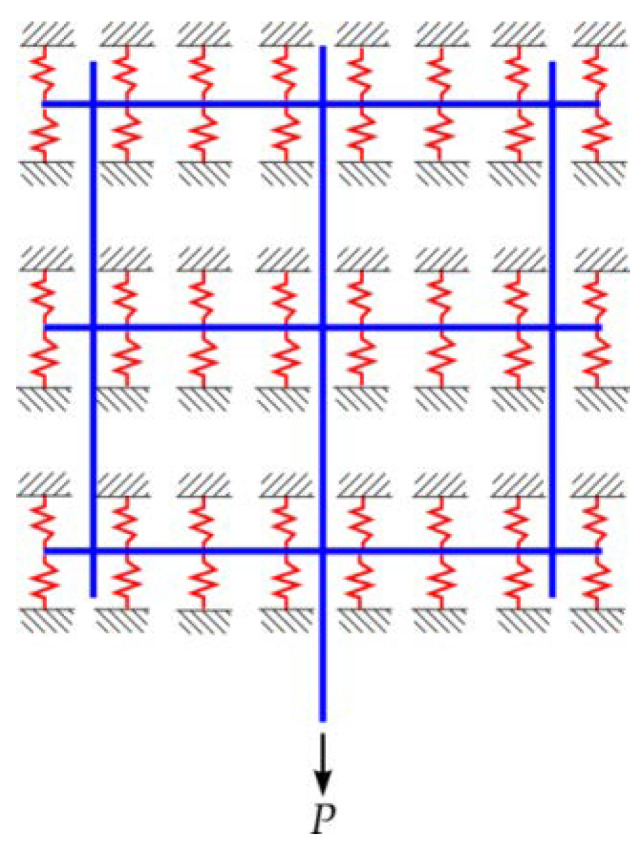
FEM analysis model.

Based on the simplified mechanical model, the two-dimensional FEM model was established using the FEM software ABAQUS. According to the stress behavior of the CFRP grid in the PCM, when the vertical CFRP grid was elongated along the axial direction under the tensile load, the horizontal CFRP grid would generate resistant action and cause apparent lateral deformation. Therefore, beam elements and truss elements were used to simulate the horizontal grid and vertical grid, respectively. Considering that the grid points of the CFRP grid have a certain rigidity, the crossing points of the horizontal and vertical grids were simulated by rigid joints. The nonlinear springs were arranged along the axis of the horizontal grids, and a nonlinear spring was arranged on the two sides of the grid points to constrain the CFRP grid. Taking the specimen C8D100, for example, the FEM model is shown in Figure 11. The spring stiffness was calculated by Equation (1), where the interface shear strength was obtained via the direct shear tests between the PCM and the concrete [26], and the tensile strength of the PCM was obtained from the material property tests (see Table 4). The critical deformation *d*_t_ and maximum deformation *d*_max_ of the nonlinear spring were 0.1 mm and 0.3 mm, respectively. The tensile strength *σ*_t_ of the PCM and interface shear strength *σ*_s_ between the PCM and the concrete were 3.21 N/mm^2^ and 4.69 N/mm^2^, respectively. Spring spacing was 2.5 mm. It was assumed that the stress distribution in the area around each spring gradually decreased, and that only half of the area around each spring plays a role in bearing stress. Thus, the adjustment coefficient was set as 0.5. The bearing capacity of springs at different locations can be obtained by substituting these parameters into Equation (1), as shown in Table 7. It can be seen that the maximum resistant actions *R*_max_ of the bottom springs were lower than those of the non-bottom springs, because the support area of the CFRP grid near the bottom loaded edge was smaller than that of the CFRP grid far away from the bottom loaded edge.

#### 5.2.2. Model Verification

**Figure 12:**
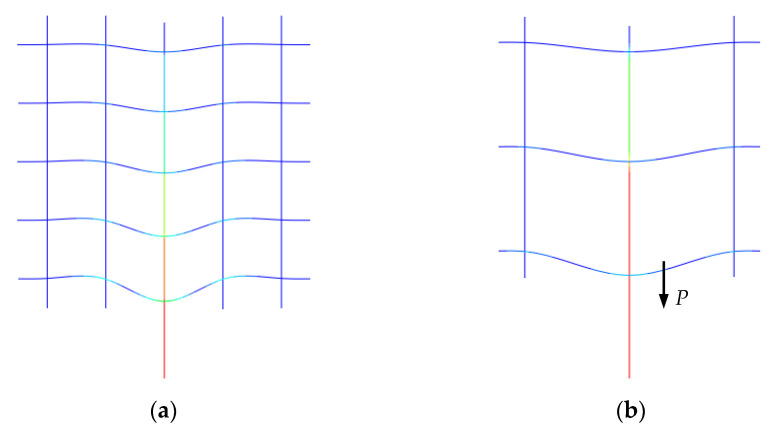
Diagram of specimen deformation. (**a**) diagram of specimens C6D50 and C8D50 deformation; (**b**) diagram of specimens C6D50 and C8D50 deformation.

**Figure 13:**
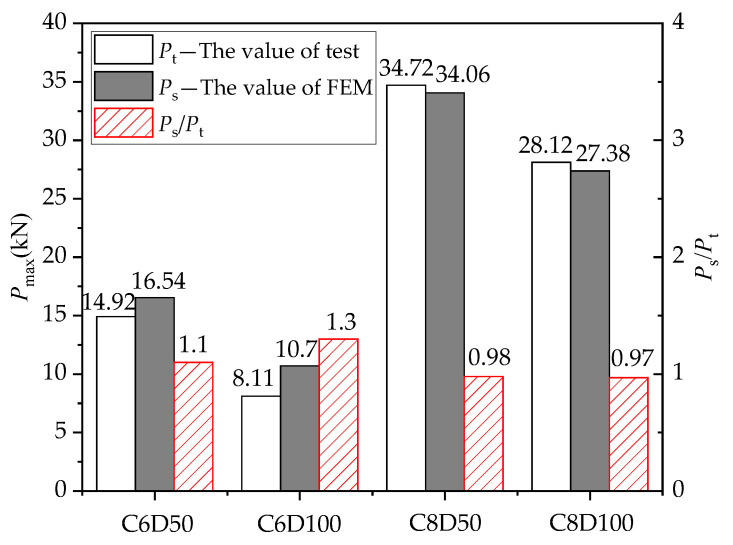
Comparison between the FEM and corresponding experimental results.

Figure 12 shows the deformation of the CFRP grid for the FEM model based on the specimen C8D100. It can be seen that the deformation of the CFRP grid near the loaded edge was larger than that of the CFRP grid far away from the loaded edge, while the tensile stress was gradually transferred from the loaded edge to the free edge. Figure 13 shows the comparison of the maximum tensile loads obtained via the pull-out tests and FEM calculations. It can be seen that the ratios of the test values to the simulation values were close to 1, indicating that the simplified interface model with a nonlinear spring system could effectively reflect the mechanical behavior of the concrete specimens with a CFRP grid–PCM reinforcing layer. There was a certain error between the ultimate load of the FEM results and the test values. The main reason for these differences was that the simplified model considered the interface interaction between the CFRP grid–PCM and the concrete, as well as the interaction between the CFRP grid and the PCM from the overall point of view, which is an equivalent simplification of the interaction of the various parts. In addition, the nodes of the CFRP grids were regarded as rigid connections, which caused the inaccurate expression of the working performance of the actual nodes under loading.

**Figure 14:**
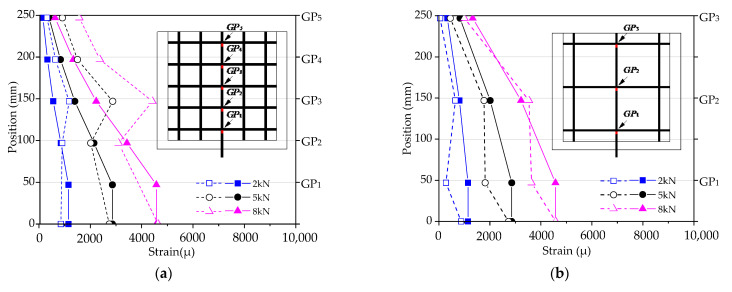

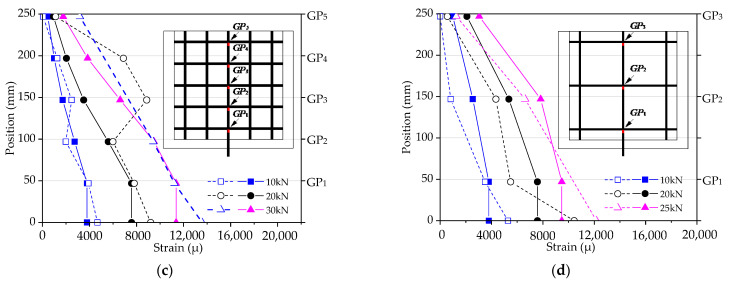
Comparison of strain distribution for vertical grids. (**a**) comparison of strain distribution for vertical grids in specimen C6D50; (**b**) comparison of strain distribution for vertical grids in specimen C6D100; (**c**) comparison of strain distribution for vertical grids in specimen C8D50; (**d**) comparison of strain distribution for vertical grids in specimen C8D100.

The FEM analysis results were compared with the test results, as shown in Figure 14. It can be observed that the pull-out test results and the FEM analysis results were almost identical in the strain distribution patterns of the tensile vertical grids; that is, the tensile strains gradually deceased from the loaded edge to the free edge. Therefore, it is reasonable to reflect the stress transfer change by the equivalent springs in the simplified mechanical model. In addition, the FEM strain values at some grid points were inconsistent with the test strain values. As shown in Figure 14a,c, the FEM strain value of specimens C6D50 and C8D50 at nodes GP1 and GP2 were essentially consistent with the test strain value; however, a discrepancy occurred at GP3. The test strain value increased suddenly at GP3, indicating that the specimen bore a large load and the force transfer form changed, which was probably related to the cracking of the mortar in the test process that was unable to be completed in the simplified simulation. In conclusion, since the simplified model considering the equivalent simplification based on reasonable analysis was the inaccurate simulation of the stress of the original specimens, the stress state of the vertical or transverse reinforcement was different from that in the test, and a certain error occurred between the FEM value of the node strain and the test value. Further investigations using a fine mechanical model considering grid point failure should be conducted in the follow-up study.

#### 5.2.3. Strain Analysis of CFRP Grids

**Figure 15:**
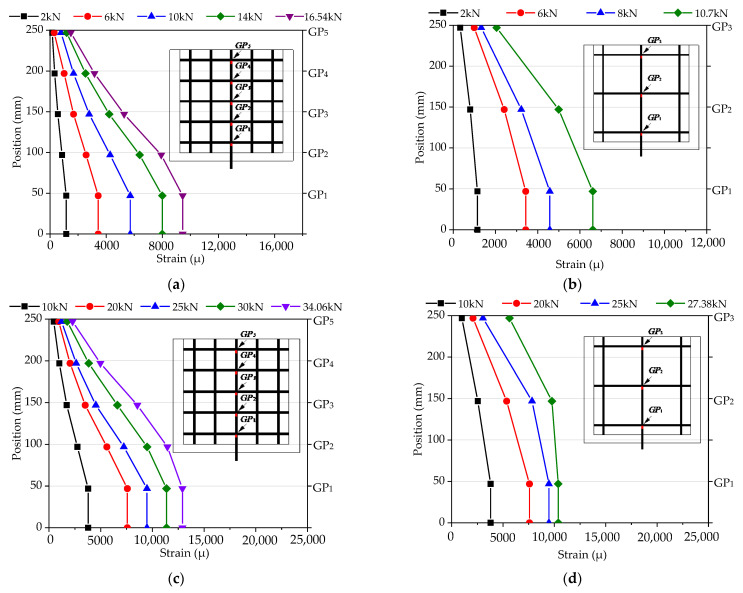
Strain distribution for CFRP grids. (**a**) strain distribution for CFRP grids in specimen C6D50; (**b**) strain distribution for CFRP grids in specimen C6D100; (**c**) strain distribution for CFRP grids in specimen C8D50; (**d**) strain distribution for CFRP grids in specimen C8D100.

Figure 15 shows the strain distribution in the FEM results to explain the stress transmission change. The results show that the trend of strain distribution on vertical grid points was almost identical for all pull-out specimens during the loading process. There were three stages in the variation of strain distribution with load: In the initial stage, the strain of the vertical grids was concentrated at the loaded edge, indicating that the load was mainly undertaken by GP1 and GP2, and the strain at GP1 was larger than at GP2, owing to the transmission mode of the vertical grid load. With the load increasing, the strain growth tendency from GP1 to GP5 (GP3) decreased, indicating that the load was gradually transferred from the loaded edge to the free edge. In the final stage, the load of specimens was close to its maximum, the spring failure expanded, and the strain at the grid points increased. With all springs failed, the specimens completely lost their carrying capacity. In addition, as shown in Figure 15a,b, comparing the strain distribution at the grid points at a certain load for C6D50 and C6D100 (i.e., 2 kN and 6 kN), it can be seen that with the upward transfer of stress, GP3 in C6D50 is less than GP2 (at the same height) in C6D100, indicating that more grid points can share the stress with small grid spacing at the identical cross-sectional area. As shown in Figure 15a,c, for specimen C6D50, at a load of 2.0 kN, the grid strain of GP4 and GP5 is close to 0. When the load reaches 10.0 kN, the strain of GP1 to GP5 decreases; due to the large cross-sectional area, the grid point strain of C6D50 is greater than that of specimen C8D50, which is consistent with the results of carrying capacity, indicating that the upper grid point of the vertical grid can effectively bear the load and improves the carrying capacity of the specimens.

#### 5.2.4. Strain Difference Analysis

**Figure 16:**
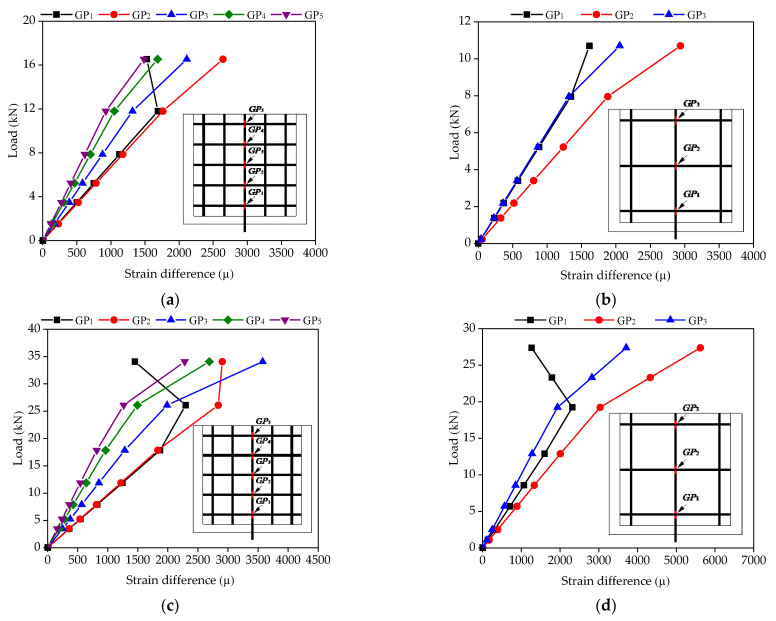
Strain difference distribution for CFRP grids. **(a**) C6D50; (**b**) C6D100; (**c**) C8D50; (**d**) C8D100.

The variation in strain difference on both sides of vertical grids in different specimens is shown in Figure 16, wherein the load–strain difference curve shows an upward tendency with the increase in pull-out load for all specimens, and the linear relationship mostly occurs in the initial stage. The farther away from the loaded edge, the smaller the strain difference, indicating that the resistance of the transverse reinforcement is smaller. As can be seen from Figure 16a,c, the strain difference at GP1 is close to that of GP2, which shows that the force is mainly undertaken by these two grid points. As the load reached a certain value (specimen C6D50 in 11.8 kN and specimen C8D50 in 17.9 kN), the strain difference of GP1 changed with the gradual failure of some springs at the grid point after the softening stage, and the load was borne by the spring at the next adjacent position. Then, with the load increasing, the force was continuously transmitted upward, and the resistance of the transverse grids further reduced until the ultimate load. As can be seen from Figure 16b,d, the overall strain difference variations of specimens C6D100 and C8D100 were effectively consistent with the others. However, the strain difference of GP1 was smaller than that of GP2 throughout the whole loaded process, due to the concentrated load at GP1 for the larger grid interval of CFRP grids, resulting in the premature failure of some springs, after which the load was transmitted to GP2, which bore the main load, even if the elastic spring near GP1 did not completely quit working.

## 6. Conclusions

In this paper, the interface bond mechanism between the CFRP grid–PCM reinforcing layer and concrete was investigated via pull-out tests, and a numerical simulation method based on the simplified mechanical model was proposed. The main conclusions can be drawn as follows:

(1) The tensile strength utilization ratio of the CFRP grid in specimen C6D50 was 81% higher than in specimen C6D100, and the tensile strength utilization ratio of the CFRP grid in specimen C8D50 was 23% higher than in specimen C8D100. Meanwhile, the maximum load of specimen C8D50 with the CR8@50-type CFRP grid was nearly 2.5 times higher than that of specimen C6D50 with the CR6@50-type CFRP grid, and the maximum load of specimen C8D100 with the CR8@100-type CFRP grid was 3.5 times higher than that of specimen C6D100 with the CR6@100-type CFRP grid. The use of a CFRP grid with a larger cross-sectional area and smaller grid interval can significantly improve the interfacial bearing capacity between the CFRP grid–PCM reinforcing layer and the concrete. Moreover, the interfacial bond behavior between the reinforcing layer and the concrete can be effectively enhanced by using an interfacial binding agent;
(2) The tensile stress in the CFRP grid deceased gradually from the loaded edge to the free edge. The tensile stress borne by the CFRP grid was transferred to the PCM by the resistant action of the horizontal grid and the bond action of the vertical grid, and then to the concrete by the interface bond between the PCM and the concrete. The interfacial bearing capacity between the CFRP grid–PCM reinforcing layer and the concrete depended on the tensile strength of the CFRP grid, the anchorage action of the CFRP grid in the PCM, and the bond behavior between the PCM and the concrete;
(3) The simplified interface model with a nonlinear spring system could effectively reflect the mechanical behavior of the concrete specimens with the CFRP grid–PCM reinforcing layer, indicating that it can be used to simulate the stress transfer modes and interface bond mechanisms between the CFRP grid, PCM, and concrete;
(4) The stress was gradually transferred from the loaded edge to the free edge through the grid action in the process of drawing the load. There was an obvious turning point in the load–strain difference curve in the CFRP grid, indicating the change in the tendency of the resistance of transverse reinforcement—that is, the stress transfer mode changes with the failure of the springs.

## Figures and Tables

**Table 1:** Types of the specimens.

Specimen	CFRP Grid Type	Grid Interval (mm)	Dimension of Concrete (mm)	Dimension of PCM (mm)
C6D50	CR6	50	300 × 270 × 90	250 × 250 × 40
C6D100	CR6	100		
C8D50	CR8	50	300 × 270 × 90	250 × 250 × 40
C8D100	CR8	100		

**Table 2:** Mixture proportions of concrete.

Specimen	G_max_ * (mm)	SL * (mm)	W/C (%)	Air Content (%)	Unit Content (kg/m^3^)			
					W *	C *	S *	G *
C6D50 C6D100	20	80	58	4.5	174	300	823	985
C8D50 C8D100	20	120	55.6	4.5	158	284	792	1099

* G_max_: maximum size of coarse aggregate; * SL: slump of concrete; * W: water; * C: cement; * S: fine aggregate; * G: coarse aggregate.

**Table 3:** Mixture *Proportions* of PCM.

Ready-Mixed Mortar (kg/m^3^)	Polymer (kg/m^3^)	Water (kg/m^3^)
1450	70	239

**Table 4:** Mechanical properties of concrete and PCM.

Material	Specimen	Compressive Strength (N/mm^2^)	Tensile Strength (N/mm^2^)	Elastic Modulus (kN/mm^2^)
Concrete	C6D50 C6D100	33.2	3.0	25.3
	C8D50 C8D100	36.7	2.87	30.1
PCM	C6D50 C6D100 C8D50 C8D100	47.1	3.21	16.8

**Table 5:** Mechanical properties of CFRP grid.

Grid Type	Cross-Sectional Area (mm^2^)	Tensile Strength (N/mm^2^)	Elastic Modulus (kN/mm^2^)
CR8	26.4	1400	100
CR6	17.5	1400	100

**Table 6:** Summary of test results.

Specimen	Maximum Load (kN)	Test Ultimate Tensile Load of CFRP Grid (kN)	Tensile Strength Utilization Ratio of CFRP Grid (%)	Failure Pattern of CFRP Grid	Failure Pattern of PCM	Interface Failure Pattern between Concrete and PCM
C6D50	14.92	28.6	52.2	The vertical grid was pulled out	No significant cracks were observed	Kept intact
C6D100	8.11		28.4			
C8D50	34.72	45.07	77.04		Cracks occurred on the surface	
C8D100	28.12		62.39			

**Table 7:** The maximum force of the spring.

Specimens	Grid Interval (mm)	Location of Spring	*R*_max_ (N)
C6D50 C8D50	50	Non-bottom	453
		Bottom	307
C6D100 C8D100	100	Non-bottom	747
		Bottom	307

## Data Availability

Data available on request due to restrictions eg privacy or ethical. The data presented in this study are available on request from the corresponding author. The data are not publicly available due to the restriction of follow-up research.
